# Removal of liquid scintillator exudates by the metal organic frameworks materials: The role of functional groups

**DOI:** 10.1371/journal.pone.0315753

**Published:** 2024-12-31

**Authors:** Jie Ren, Peng Wang, Aotian Gu, Chunhui Gong, Kaiwei Chen, Ping Mao, Yan Jiao, Kai Chen, Yi Yang

**Affiliations:** 1 Jiangsu Key Laboratory of Chemical Pollution Control and Resources Reuse, School of Environmental and Biological Engineering, Nanjing University of Science and Technology, Nanjing, China; 2 National & Local Joint Engineering Research Center for Mineral Salt Deep Utilization, Key Laboratory for Palygorskite Science and Applied Technology of Jiangsu Province, School of Chemical Engineering, Huaiyin Institute of Technology, Huaian, China; 3 Collaborative Innovation Center of Atmospheric Environment and Equipment Technology, Jiangsu Key Laboratory of Atmospheric Environment Monitoring and Pollution Control (AEMPC), Nanjing University of Information Science & Technology, Nanjing, China; Faculty of Science, Alexandria University, EGYPT

## Abstract

The leakage of Liquid scintillator exudates has brought potential harm to the environment. Attributed to the large specific surface area and high modifiability, high-performance adsorbents based on metal-organic frameworks (MOFs) can effectively remove organic pollutants. In this work, we use different functional groups to prepare the material of UIO-66(Zr). These materials were used to remove dimethyl sulfoxide (DMSO) from water, which is considered a typical liquid scintillator exudate. The results showed that the UIO-66-NH_2_ (154.3 mg/g) exhibited better adsorption performance compared to the UIO-66-OH (39.5 mg/g) and UIO-66-COOH (105.8 mg/g) for the removal of DMSO. Upon examining the adsorptive abilities of various samples of different UIO-66-NH_2_ samples, it was observed that the material’s ability to adsorb is in a direct relationship with the -NH_2_ group concentration present in the substance, as evidenced by a correlation coefficient *R*^2^ of 0.99. Simultaneously, in the low concentration of environment, the samples of UIO-66 which load NH_2_ groups shows high removal effectiveness of over 90%. The adsorption capacity of the prepared materials was little affected by the complex water quality conditions and different initial pH values (between 4~10). Furthermore, the material has good reusability and adsorption capacity over five cycles, and slight zirconium release (< 5%). This optimal material showed significant removal capacity for DMSO. In conclusion, this work presents insight into the construction of advanced adsorbents for the removal of liquid scintillator exudates that have high adsorption capacity and strong potential for DMSO removal.

## Introduction

The liquid scintillation detectors have been extensively developed and utilized in the electronics industry due to their high spatial resolution in several years, angular independence, good dose response and energy response, and real-time readout. However, the preparation process of liquid scintillator involves a large amount of organic matter [1–5]. At the same time, Extensive use and disposal of liquid scintillators lead to a large amount of organic matter entering the water environment [6, 7]. Although most Liquid scintillator exudates cannot be dispersed and completely dissolved in water, there are still some special Liquid scintillator exudates such as aromatic hydrocarbons and alkanes molecules that have good water solubility and cause greater harm to the environment [8–10]. As a common exudate in the fields of liquid scintillator and other chemical materials, Dimethyl sulfoxide (DMSO) is commonly used because of its unique physical and chemical properties [11, 12]. Nevertheless, the structure and containment of DMSO has a huge potential harm to the environment, and affect the stability of the ecosystem. Therefore, the research on the removal of Liquid scintillator exudates such as DMSO is expected to promote the green development and application of liquid scintillator [13–15].

Since MOF-9 was first defined in 1995, more than 20,000 different MOFs have been discovered and applied in diverse fields such as adsorption, catalysis and energy storage [16]. Due to their relatively large specific surface area and tunable pore structure, a variety of MOFs could be modified into excellent adsorbents by different strategies, which has been demonstrated in a large number of researches on the adsorption of pollutants in water [16, 17]. For example, Zr (IV)-based MOFs are highly hydrophilic and stable, and therefore have great potential for application in water purification [18–20]. Lin et al. investigated the adsorption properties of MOFs materials in aqueous medium by employing Zr(IV) materials for functional group loading, which resulted in the adsorption of NSAIDs in water [21]. In addition, Yuan et al. also prepared a metal framework material called ZIF-300, and they successfully deposited ZIF-300 on an aluminum base using the secondary growth method to obtain a ZIF-300 membrane, which has a high filtration rate and also demonstrates excellent operational stability for the removal of heavy metal ions [22]. Yao et al. investigated the performance test of MOFs materials for the removal of hydrophilic dyes in water, and they successfully prepared a novel membrane material, named UiO-66-Urea-based flexible membrane, which showed good separation ability for mixed dyes in complex water quality [23]. Therefore, adsorption using MOFs materials is a good mean in the removing program of organic contaminants in water [24–26].

This study investigated the feasibility of MOFs materials for the removal of liquid scintillator pollutants from water bodies. DMSO was taken as a typical pollutant in the experiments, and three kinds of UIO-66 with different functional groups were synthesized by amino, hydroxyl and carboxyl modifications, and their adsorption and removal properties on DMSO, a typical pollutant in liquid scintillator, were analyzed by experiments. The results showed that the amino group was the key group for DMSO removal. Meanwhile, the content of amino groups in the material is linearly related to the adsorption capacity of pollutants. We believe that MOFs can be used as one of the options for the removal of Liquid scintillator exudates, and we speculate that materials containing amino adsorption sites have great application potential for the adsorption and removal of Liquid scintillator exudates, which will promote the green production and application of Liquid scintillator.

### Experimental

#### Chemicals and characterizations

The use of all reagents in this experiment is described in SI.

#### Characterizations

For characterization, scanning electron microscopy (SEM), Fourier transform infrared spectroscopy (FT-IR), and X-ray diffractometry (XRD) were used to measure the morphology and functional groups of the MOFs materials, and in the analysis of the porous structure and specific surface area of the MOFs materials used (BET) in order to study the porous structure of UIO-66-X.

## Synthesis of materials

### Synthesis of UIO-66 with functional groups

The adsorbent material was slightly modified on the existing synthesis strategy [27]. In a typical synthesis, 30 mL dimethylformamide (DMF) that contain 20 mM zirconium tetrachloride (ZrCl_4_) was sonicated for 1 h. Then 0.867 mmol of crosslinker was added gradually during stirring and stirring was continued for 15 minutes. The cross-linkers were 1,2,4,5-benzenetetracarboxylic acid, 2-hydroxyterephthalic acid, and 2-aminoterephthalic acid, which were used for the synthesis of adsorbent materials functionalized with different functional groups. Subsequently, the mixed solution was then poured into a 50 mL size Teflon autoclave for 24 hours with the temperature set at 120°C. The material prepared in the reactor was washed several times with DMF as detergent, and the washed material was immersed in deionized water and washed again, and the finally obtained materials were named as UIO-66-X, Amino-modified material named UIO-66-NH_2_, Hydroxyl-modified material named UIO-66-OH, the carboxyl-modified material is named UIO-66-COOH.

### Synthesis of UIO-66-NH_2_ with different -NH_2_ content

30 mL dimethylformamide (DMF) that contain 20 mM zirconium tetrachloride (ZrCl_4_) was sonicated for 1 h. Then 2-aminoterephthalic acid was added to the mixed solution and stirring was continued with the concentration of 2-aminoterephthalic acid added to the mixed solution being 0.433, 0.866, and 1.299 mmol, respectively. stirring was then continued for 15 min. Subsequently, A 50 mL size Teflon autoclave was used to place the stirred mixed solution, setting the temperature to 120°C for 24 hours. The materials obtained in the reactor were rinsed several times using DMF and then soaked in deionized water, and the material prepared using 0.433 mmol of modifier was named UIO-66-NH_2_-1, the material prepared using 0.866 mmol of modifier was named UIO-66-NH_2_-2, and the material prepared using 1.299 mmol of this property was named UIO- 66-NH_2_-3.

### Adsorption experiment

#### Batch experiments

Experimental quantification of DMSO adsorption involved introducing 10 milligrams of various adsorbent materials into 100 milliliters of DMSO containing varying concentration levels. The reaction mixture’s starting pH was set to 7.0 using a 0.1 M solution of HCl or NaOH, and continuous agitation was maintained at a rate of 200 revolutions per minute for a duration of 12 hours. For the measurement of the equilibrium concentration of DMSO this experiment was performed using gas chromatography. The relevant adsorption isotherm equations are shown in Supporting.

#### Adsorption kinetics

To examine the adsorption behavior of DMSO over time, the analogous procedure was carried out in the subsequent sequence of actions. Add approximately 10 mg of UI0-66 to 100 ml of dimethyl sulfoxide solution (50 mg/L). Initially, the reaction mixture’s pH was calibrated to a neutral value of 7.0 by adding either 0.1 M HCl or NaOH, while the mixture was agitated continuously at a pace of 200 revolutions per minute for a period of half a day. Periodically, samples of 1 ml were extracted from the mixture to monitor the remaining concentration of the intended pollutants. The adsorption kinetics equations were shown in the Supporting.

#### Effect of the initial pH

Examining the influence of starting pH on adsorption efficacy, the reaction mixture’s beginning pH was modified between 4.0 and 10.0 with the use of 0.1 M HCl/NaOH, while maintaining experimental parameters as outlined in section 2.4.2.

### Influences of inorganic negatively charged ions and organic compounds occurring in nature

To examine the impact of simultaneous presence of substances in water-based environments on the adsorptive capacities of various materials, assorted levels of inorganic anions (sulfate, nitrate, chloride, bicarbonate), humic acid, and bovine serum albumin were introduced into the mixtures. Additional experimental parameters mirrored those outlined in section 2.4.2. The mixtures were agitated at ambient temperature for 12 hours prior to measuring the concentration of remaining pollutants.

### Evaluation of recyclability

After the adsorption experiments, samples of UIO-66-Founctional group were treated with 50 mL of methanol contained 5 mL of 0.1 M NaOH for 6 h. The processed specimens underwent a second round of vacuum drying before the adsorption tests were replicated, with this cycle of experiments being conducted a total of ten times.

### Experiment on actual water purification

A mimicry of contaminated water was created by supplementing a local water specimen from Nanjing’s Zixia Lake with extra DMSO (5.0 mg/L), while the remaining experimental procedures aligned closely with those outlined in section 2.4.2. Table 1 provided a comprehensive comparison of the chemical constituents in the artificial polluted water prior to and subsequent to the adsorption involving UIO-66-NH_2_.

**Table 1:** Solution chemistry of the simulated polluted water before and after adsorption by UIO-66-NH_2_.

Species	Concentrations (mg/L)	Concentrations (mg/L)
	before adsorption	after adsorption
K^+^	2.168	2.034
Na^+^	4.684	4.413
Ca^2+^	16.231	16.007
Mg^2+^	5.412	5.153
Pb^2+^	0.286	0.255
Cu^2+^	0.578	0.542
DMSO	5.0	0.937

## Discussion

### Characterization of UIO-66-X

**Fig 1:**
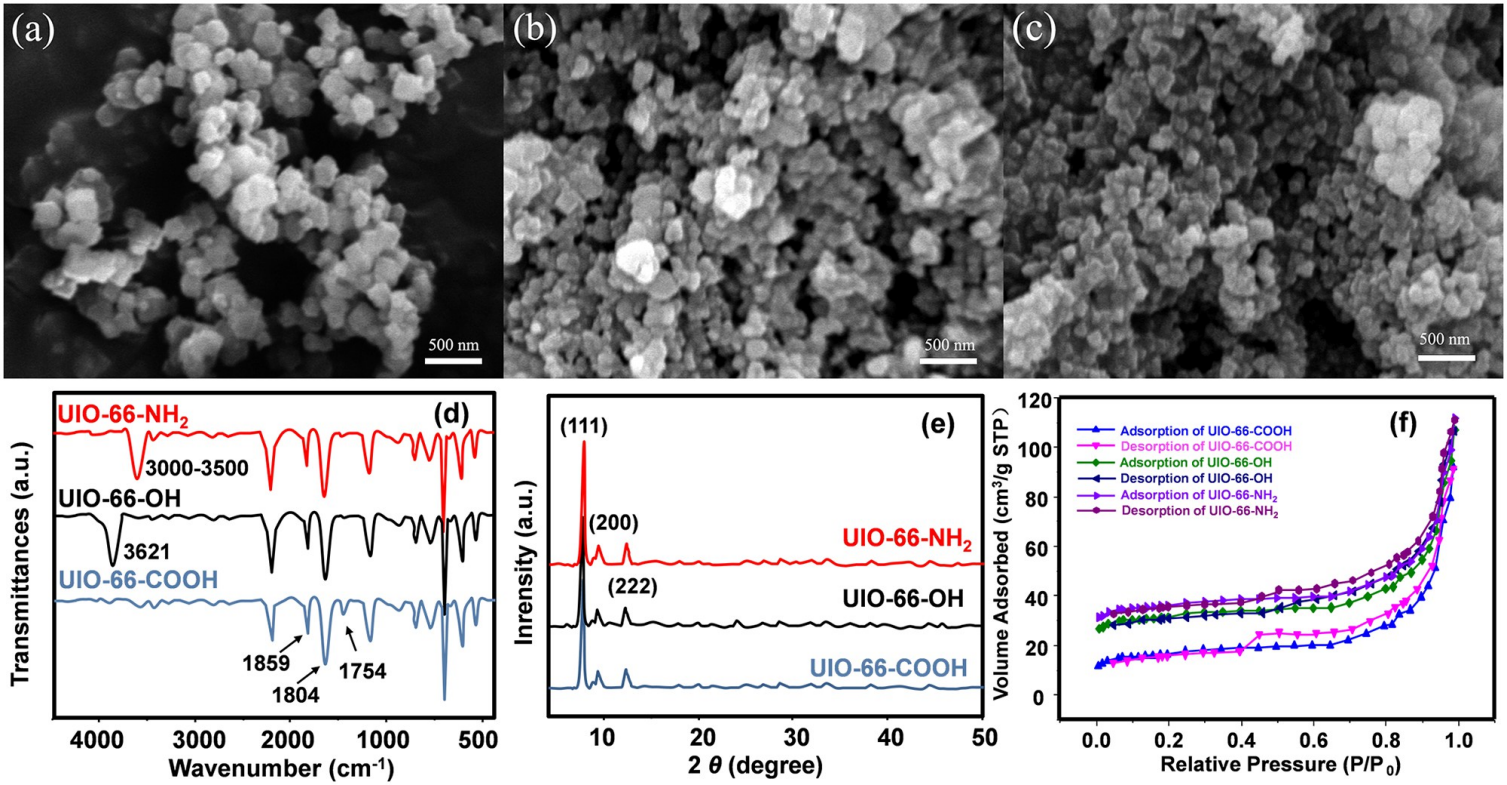
Displays scanning electron microscopy (SEM) visualizations for UIO-66-NH_2_ (a), UIO-66-OH (b), UIO-66-COOH (c), alongside Fourier-transform infrared (FT-IR) spectroscopy readings (d), X-ray diffraction (XRD) patterns (e), and nitrogen adsorption-desorption curves for UIO-66-X (f).

Initially, the prepared specimens’ configuration and form were determined using scanning electron microscopy. Examination of the SEM photographs in Fig 1a–1c reveals that the morphology of all three samples were irregular nanoparticles with the average size of the particles of about 50 nm, and no significant differences were observed. This means that the different functional groups did not affect the microstructure of UIO-66 materials [28].

In this experiment, FTIR and X-ray diffraction were used to characterize the chemical structure of UIO-66-X samples, and in Fig 1d, it can be seen that the UIO-66-NH_2_-X samples exhibit characteristic peaks of anhydride stretching corresponding to the cross-linker at 1859 cm^-1^, which can be seen at 1804 cm^-1^ as well. In the interim, the distinctive peak at 3621 cm^-1^, indicative of the -OH stretching vibration, is exclusively observed in samples containing the -OH group. Conversely, the -NH_2_ group is solely responsible for peaks in the range of 3000–3500 cm^-1^, and the distinct peak at 1754 cm^-1^, which is characteristic of the -COOH group, is found only in UIO-66-COOH. These findings confirm the effective synthesis of three distinct materials, each modified with a separate functional group [29, 30].

Fig 1e illustrates that the X-ray diffraction (XRD) profile for the synthesized specimens matches the UiO-66 standard. Notably, the distinctive peaks occurring at 7.4°, 8.5°, 14.8°, 17.1°, 22.3°, 25.7° and 30.8° in the theoretical XRD spectra of unmodified metal-organic frameworks (MOFs) correspond to the crystal planes (111), (200), (222), (400), (511), (600), and (711), in that order. It could be seen that the three different samples have these peaks, which indicates that the different functional groups do not affect the microstructure of the UIO-66 material [31, 32].

As illustrated in Fig 1f, the porous structure of UIO-66-X was investigated by N_2_ adsorption-desorption experiments. The adsorption isotherms revealed that the BET specific surface areas for UIO-66-NH_2_, UIO-66-OH, and UIO-66-COOH are, respectively, 1200, 1000, and 642 square meters per gram. S1 Table in S1 File showed the pore properties of UIO-66-X with average pore sizes of 8.54, 7.21, and 6.87 nm and corresponding pore volumes of 0.675, 0.647, and 0.576 cm^3^/g, respectively.

### DMSO adsorption batch experiment

**Fig 2:**
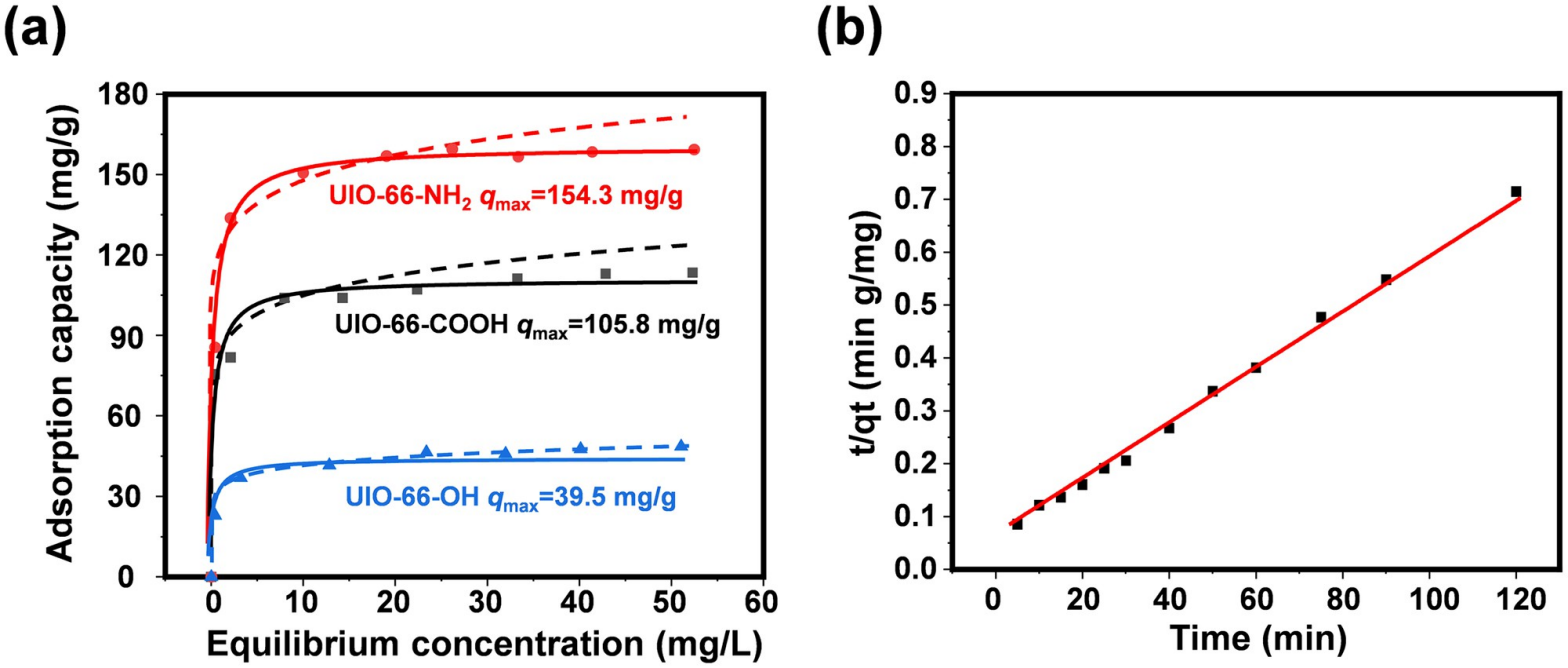
Presents the adsorptive capability of UIO-66-X for DMSO (a), along with the kinetic data of DMSO adsorption by the UIO-66-NH_2_ sample (b).

Fig 2a shows the DMSO adsorption isotherms for different functional UIO-66 samples. Referencing S2 Table in S1 File, it is apparent that fitting of all experimental observations adhered to both the Langmuir and Freundlich equations. However, the Langmuir fit prevailed over the Freundlich in terms of fit quality, as evidenced by significantly higher R2 values. Moreover, the peak adsorptive efficacy of DMSO on UIO-66-NH_2_, UIO-66-OH, and UIO-66-COOH was recorded at 164.48 mg/g, 105.8 mg/g, and 39.5 mg/g, respectively. The findings demonstrated a marked discrepancy in DMSO adsorption efficiency among diverse functional groups, leading to the choice of UIO-66-NH_2_ as the adsorbent for further testing. In addition, the parameters derived from the Freundlich isotherm model revealed a 1/n ratio within the optimal range of 0.1 to 0.5, signifying a robust affinity between DMSO and UIO-66-NH_2_ [33].

**Fig 3:**
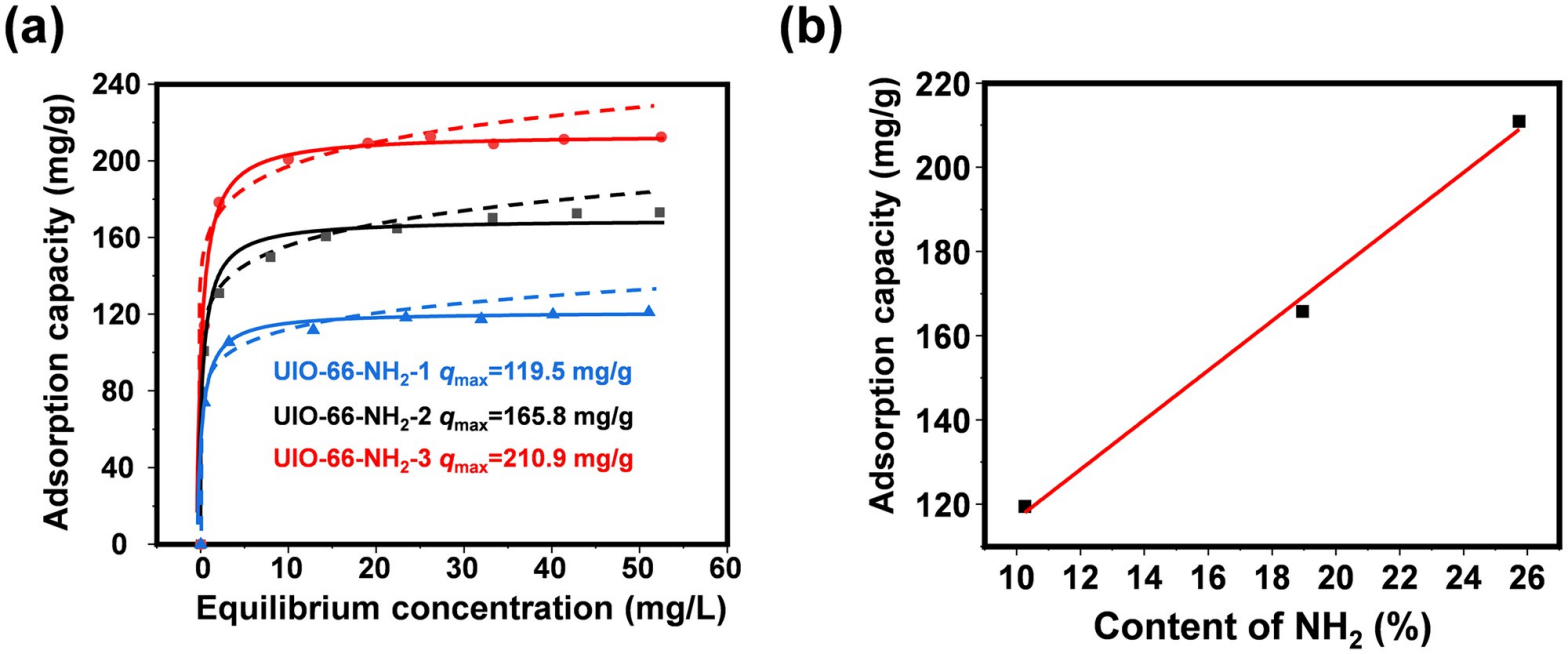
Displays the DMSO adsorption capability of UIO-66-NH_2_-X in part (a), and part (b) illustrates the correlation between the amount of -NH_2_ present in UIO-66-NH_2_ variants and their adsorptive efficacy.

Subsequently, the kinetics of DMSO adsorption on the UIO-66-NH_2_ specimen were assessed and delineated in Figs 2b and 3, S1 Fig in S1 File. In addition, all these data were fitted with pseudo-first-order. The experimental results align more closely with the pseudo-second-order kinetic model, as evidenced by superior *R*^2^ values, when compared to first-order kinetics (Eq. (S4)). Within the range of absorbent substances tested, UIO-66-NH_2_ specimens demonstrated the greatest uptake of DMSO, reaching a maximum of 165.4 mg/g, a figure that closely parallels the findings yielded by the adsorption isotherm studies. In addition, all the UIO-66-X samples achieved 100% of their maximum adsorption capacity within the first 30 min.

The adsorption kinetics, detailed in S3 Table in S1 File, demonstrated a fit with the intraparticle diffusion model, as denoted by equation S6. The presence of two distinct linear areas within the graph suggests that the DMSO uptake by UIO-66-X occurs in a biphasic manner. Initially, the specified contaminant particles migrate away from the adsorbent to adhere to its exterior, followed by a progressive movement of these contaminants into the interior of the adsorbent material. Additionally, it was noted that the initial phase of DMSO capture proceeded at a swifter pace compared to the subsequent phase, suggesting that the external mass transfer rate surpasses the internal diffusion speed within the adsorbent. Based on the data present in the scholarly works, it is possible that the elevated levels of the specific contaminant in the reactive mixture may account for this [34, 35].

### Adsorption experiment by different content of NH_2_

According to the above results, it can be observed that UIO-66-NH_2_ has excellent adsorption performance for DMSO. Therefore, we consider that the -NH_2_ was the key functional groups in the removal of DMSO.

Afterwards, we also prepared three kind of UIO-66-NH_2_ adsorbents with different -NH_2_ contents through the regulation of cross-link agents for the DMSO removal, with the content of -NH_2_ was calculated by elemental analyzer, one can see that the content of -NH_2_ in UIO-66-NH_2_-X was 10.57%, 18.25%, 24.68%, respectively. Subsequently, we assessed the sorptive capabilities of UIO-66-NH_2_-X (Fig 3a), with each outcome being modeled in accordance with the Langmuir and Freundlich isotherms, the details of which were presented in S4 Table in S1 File. It can also be observed that all data have larger *R*^2^ values when fitted to the Langmuir model compare to Freundlich model. S4 Table in S1 File illustrates that the peak adsorptive ability of the UIO-66-NH_2_-X specimens for Langmuir DMSO reaches values of 119.5, 165.8, and 210.9 mg/g, respectively. Furthermore, based on the calculation results of -NH_2_ content, we have replotted the data to evaluate the constitutive relationship between adsorption performance and -NH_2_ content. As shown in Fig 3b, it is demonstrated that the ability of UIO-66-NH_2_ composites to adsorb DMSO progressively enhances in relation to the rise in the -NH_2_ concentration, signifying that the structural and compositional modifications of the composites can systematically govern the adsorptive potential for DMSO.

**Fig 4:**
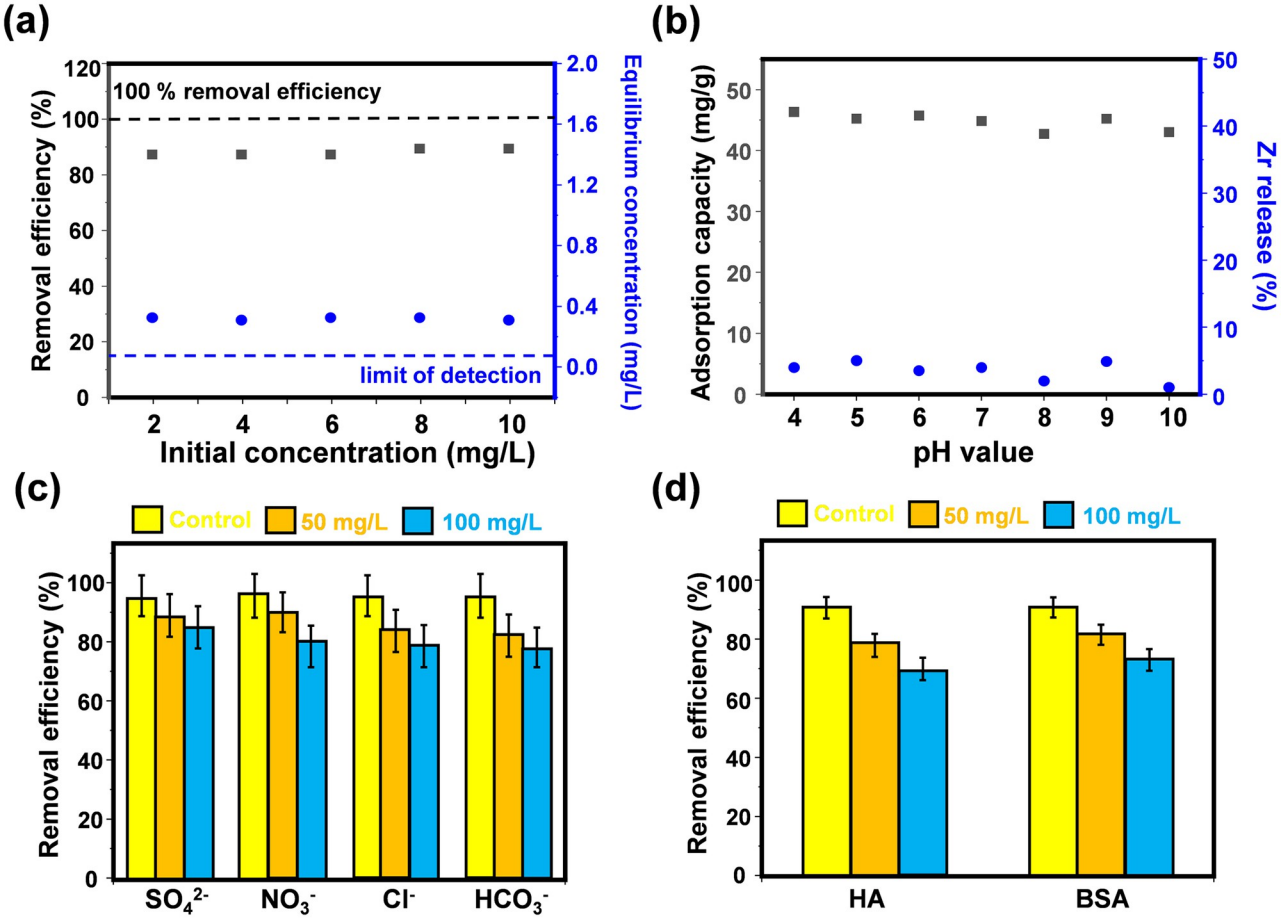
The effectiveness of DMSO elimination and its subsequent equilibrium levels following UIO-66-NH_2_-3(a) adsorption, the adsorptive prowess and zirconium leaching from UIO-66-NH_2_-3 in the presence of differing pH levels (b), and the impact of both anions (c) and organic compounds (d) on the capability of UIO-66-NH_2_-3 to purge DMSO.

Furthermore, examining how UIO-66 samples adsorb at minimal DMSO levels bears greater relevance and significance given the substance’s limited solubility in water and its typically low presence in real-world water systems. Notably, the equilibrium DMSO concentration post-adsorption by UIO-66-NH_2_-3 dropped beneath 0.5 mg/L in scenarios where the DMSO concentration was under 10 mg/L. Consequently, using an identical quantity of adsorbent (0.1 g/L), the adsorptive behavior of DMSO on UIO-66-NH_2_-3 was reassessed at varying starting concentrations. Fig 4a and S2 Fig in S1 File illustrate the efficacy of DMSO eradication across these concentrations and the adsorption capacity, respectively. Furthermore, S3 Fig in S1 File presents both the kinetic profile of DMSO adsorption and the application of the pseudo-second-order kinetic model. From which one can see that that the UIO-66-NH_2_-3 could remove nearly 90% DMSO under various initial concentration, with the DMSO equilibrium concentration was less than 0.5 mg/L.

Concurrently, it was observable that the outcomes aligned more closely with the predictions of the pseudo-second-order model. Furthermore, the estimated adsorption capacities at DMSO equilibrium, derived from the fitted pseudo-second-order model data, displayed greater concordance (18.62 for 2, 36.54 for 4, 53.97 for 6, 71.35 for 8, and 90.24 mg/L for 10 mg/L, correspondingly) when juxtaposed with the actual empirical measurements (18.12 for 2, 37.14 for 4, 54.67 for 6, 72.17 for 8, and 91.25 mg/L for 10 mg/L, respectively).

### Adsorption experiment by different content of NH_2_ in complex water environment

Furthermore, the influence of varying starting pH levels on the rate at which DMSO was desorbed was similarly assessed [36]. Fig 4b clearly shows that UIO-66 can maintain the removal of DMSO around 42.6 mg/g in the pH range of 4~10 when the concentration of DMSO and the dosage of adsorbent were 5 mg/l and 0.1 g/l. The data in S4 Fig in S1 File also show that the removal of DMSO was close to 90% over the pH range of 4 to 10, whereas the adsorption of DMSO slightly inhibited under strong alkaline conditions. This slight inhibition may be attributed to the large number of hydroxide anions in the solution hindering the reaction between -NH_2_ and DMSO. In addition, the data indicate that sample UIO-66-NH_2_-3 has the highest pH stability among all adsorbents. At the same time, the influence of starting pH on how many times the UIO-66-NH_2_-3 samples could be reused was evaluated by measuring both the percentage of zirconium that detached from the solution and the concentration of zirconium that was dissolved (mg/L), as depicted in Fig 4b and S5 Fig in S1 File. It could be seen that a negligible release of Zr (< 10%) and Zr leaching (< 1 mg/L) in a wide pH range from 4 to 10.

Aside from how starting pH levels influence adsorption, various components present in environmental water sources, such as inorganic ions (sulfate, chloride, nitrate, and bicarbonate), humic acid (HA), and bovine serum albumin (BSA), vie with dimethyl sulfoxide, the compound of interest, for adsorptive sites, thus impacting its extraction from the water [37, 38]. Consequently, to assess the selectivity of the UIO-66-NH_2_-3 in eliminating DMSO, the study examined the ramifications of two distinct doses of these competing ions, HA, and BSA, administered at concentrations of 50 mg/l and 100 mg/l. The concentrations of the two types of HA and BSA used are much higher than those in general surface water, but is similar to the range of anions found in typical wastewater [39, 40]. Fig 4c and 4d showed the removal of DMSO by UIO-66-NH_2_-3 (0.1 g/L) with/without the addition of inorganic anions, HA and BSA into the reaction solution. Without the presence of any contaminants, the DMSO uptake potency for UIO-66-NH_2_-3 stood at 44.85 milligrams per gram. Meanwhile, it could be observed that the variation of DMSO removal by UIO-66-NH_2_-3 was within 10% after the addition of various environmental matrices to the experimental system. The data also showed the removal of DMSO by UIO-66-NH_2_-3. The results showed the removal of DMSO was close to 90% in pure solution, while the removal of DMSO varied in the range of ± 3 mg/L under the influence of inorganic anions, HA and BSA. These results indicate that UIO-66-NH_2_-3 exhibits excellent selective adsorption capacity for DMSO.

### Stability and real application

**Fig 5:**
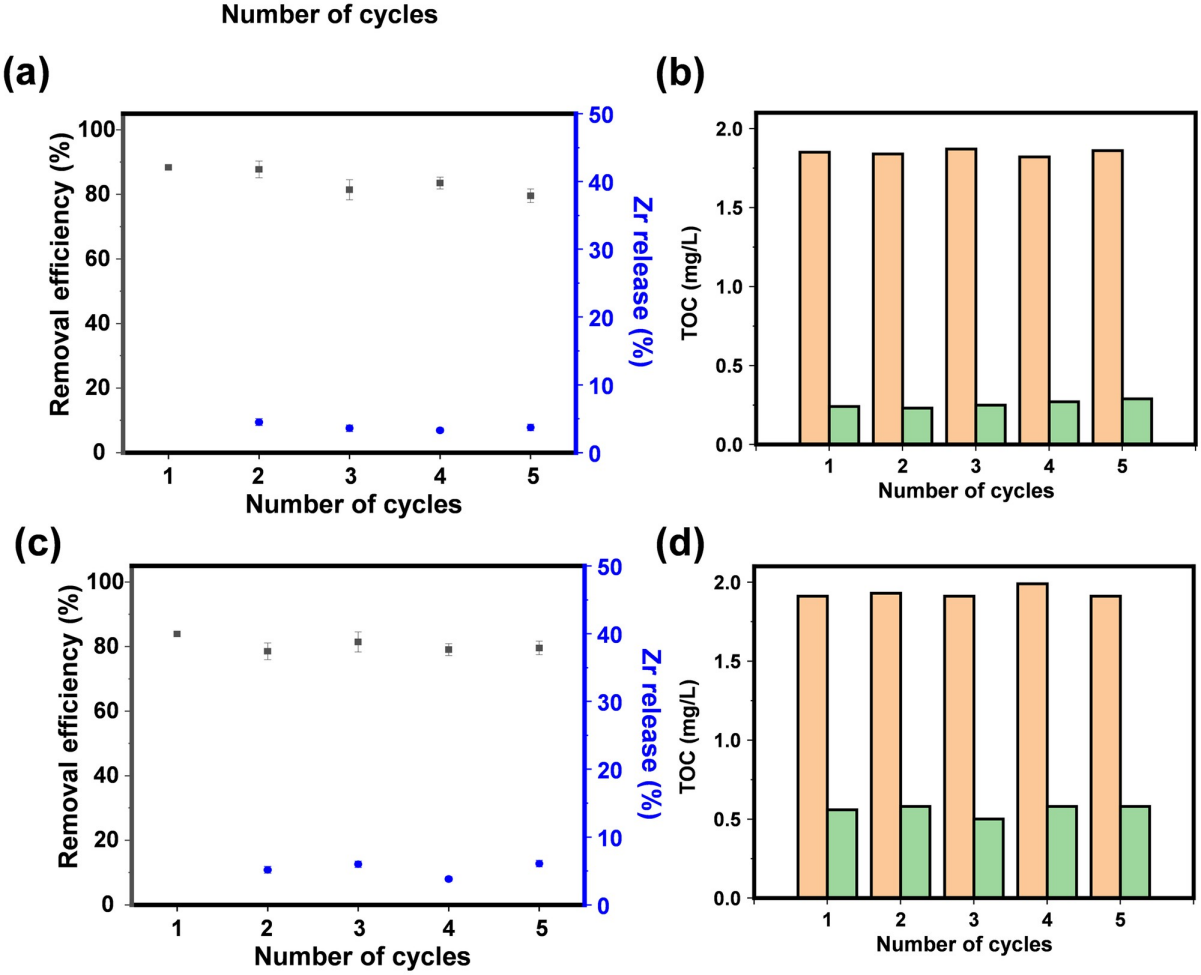
The removal efficiency and Zr release(a), TOC changes of UIO-66-NH_2_-3 towards DMSO in pure water over five cycles (b), the removal efficiency and Zr release (c), TOC changes of UIO-66-NH_2_-3 towards DMSO in real water over five cycles (d).

Following their use, the UIO-66-NH2-3 specimens underwent a brief methanol rinse accompanied by sonication, after which their adsorptive abilities and structural integrity were evaluated. The material’s consistency in adsorption was assessed through five iterative cycles, with both the efficiency of contaminant removal and the adsorption prowess across these cycles being depicted in S7a Fig in S1 File. One can see that the UIO-66-NH_2_-3 could maintain excellent removal efficiency (> 85%) and adsorption capacity (> 40 mg/g) over five cycles. Moreover, one can see the negligible Zr release (< 5%) and Zr leaching (< 1 mg/L, S7b Fig in S1 File), highlights UIO-66-NH_2_-3’s impressive stability. Furthermore, to assess UIO-66-NH_2_-3’s capacity for adsorption in multiple water samples with DMSO, the reaction solution’s total organic carbon (TOC) levels were determined. As shown in Fig 5b, adsorption of DMSO by UIO-66-NH_2_-3 was able to remove 85% to 92% of TOC in five cycles.

Post five repetitive testing iterations, the UIO-66-NH_2_-3’s microstructural integrity and surface chemistry were analyzed using techniques such as Scanning Electron Microscopy (SEM), Fourier-Transform Infrared Spectroscopy (FTIR), and X-ray Diffraction (XRD). The SEM images revealed that, even after undergoing five cycles, the rejuvenated UIO-66-NH_2_-3 preserved its original microscale form. The FTIR spectrum in S8b Fig in S1 File demonstrated the chemical composition of regenerated UIO-66-NH_2_-3 remained intact during the adsorption process. However, it could be observed from the XRD pattern in Fig. The repeated cycling tests resulted in lower crystallization levels of the regenerated specimens, thereby confirming that UIO-66-NH_2_-3 retains its molecular configuration and adsorptive efficacy.

Genuine samples of water were gathered from Nanjing’s indigenous Zixia Lake, into which a specific amount of DMSO was introduced. Additionally, the prepared specimens underwent assessment to determine their adsorption ability and the effectiveness with which they removed impurities, repetitively tested across five iterations. It could be observed that UIO-66-NH_2_-3 displayed superior adsorption capacity and selectively towards the DMSO, with the good stability (Zr release < 5%). In addition, we found that UIO-66-NH_2_-3 was able to reduce TOC from nearly 2.0 to below 0.6 mg/L after five consecutive runs.

Then we have listed the chemical composition of the simulated real wastewater before and after adsorption by UIO-66-NH_2_ as shown in Table 1. One can see that the DMSO concentration decreased from 5.0 to 0.937 mg/L while concentrations of other species were maintaining a stable level. All these results demonstrate the remarkable potential of UIO-66-NH_2_-X for DMSO removal from real water bodies.

## Conclusion

In the present research, we initially document the employment of Metal-Organic Frameworks (MOFs) for the adsorption of DMSO, a compound found in Liquid scintillator effluents, from aqueous environments. Experimental data indicate that UIO-66 variants tailored with various functional groups demonstrate disparate absorption efficacies. Specifically, UIO-66-NH_2_ showed superior adsorptive capabilities and the most pronounced performance metrics in comparison to its counterparts, the UIO-66-NH_2_-X samples. The correlation between varying amounts of -NH_2_ and adsorption efficiency is affirmative, indicating that the adsorption capability can be modulated through the alteration of the -NH_2_ concentration within the substance. In summary, we believe that MOFs-based materials, especially -NH_2_ containing materials, can be used as effective adsorption materials for DMSO, and speculate to other -NH_2_ containing materials. We believe that we have found a reasonable method and material for the removal of Liquid scintillator exudates materials, and it is expected to be popularized and applied on a large scale.

## Supporting information

S1 File (DOCX)
