# Supplementary material for: Removal of liquid scintillator exudates by the metal organic frameworks materials: The role of functional groups
Source: PLoS One. 2024 Dec 31;19(12):e0315753. doi: 10.1371/journal.pone.0315753 (PMC11687881; doi:10.1371/journal.pone.0315753)
Supplement: S1 File — (DOCX) [file pone.0315753.s001.docx]

**Supplementary material for**

**Removal of Liquid scintillator exudates by the metal organic frameworks materials: the role of functional groups**

**Materials**

Unless otherwise specified, all chemicals used in this study were of analytical grade. acid (HCl), sodium hydroxide (NaOH), *N*,*N*-dimethylformamide (DMF), methanol and ethanol were all purchased from Sinopharm Chemical Reagent Co., Ltd. Pentafluorophenol, Zirconium Tetrachloride, 2-hydroxyterephthalic acid, 2-aminoterephthalic acid, 1,2,4,5-benzenetetracarboxylic acid, were purchased from Shanghai Aladdin Bio-Chem Technology Co., Ltd. All working solutions were prepared by Milli-Q water with a resistivity of 18.25 MΩ·cm, and stock solutions were kept in dark at 4 ^o^C.

**Characterizations**

**Fourier transform infrared spectroscopy (FT-IR)**

FT-IR spectra were collected on a Thermo Scientific Nicolet Is5 Spectrometer. The samples were loaded on a diamond ATR crystal plate. The spectra were generated by cumulating 32 scans at a resolution of 2 cm^-1^, with scanning wavelengths from 500 cm^-1^ to 4000 cm^-1^.

**Electron microscopy**

Scanning electron microscopy (SEM) images a were collected using a Hitachi S-4800 operating at 25 kV.

**Brunauer-Emmett-Teller (BET)**

S_BET_ is the Brunauer–Emmett–Teller (BET) surface area (in units of m^2^/g) calculated from the N_2_ adsorption isotherm; P and P_0_ are the equilibrium and saturation pressures of N_2_ at 77 K, respectively. The Barret–Joyner–Halenda (BJH) method was used to estimate the pore volume and pore diameter from the adsorption isotherm.

**The related equations of adsorption isotherms**

The adsorption amount *q*_e_ (mg/g) is calculated as via Eq. S1:

$q\text{e}=\left[ \left( C\text{0}-C\text{e} \right)V \right]/m$ Eq. S1

where *C*_0_ and *C*_e_ (mg/L) are the initial and equilibrium concentrations of pollutants, *V* (mL) is the volume of the solution and *m* (mg) is the weight of the adsorbent.

The data are fitted to both Langmuir (Eq. S2) and Freundlich models (Eq. S3):

$\text{C}\text{e}/\text{q}\text{e} = \text{C}\text{e}/\text{q}\text{m} + 1/(\text{K}\text{L} \text{q}\text{m})$ Eq. S2

$\ln\text{q}\text{e} = ln\text{K}\text{F} +( ln\text{C}\text{e})/n$ Eq. S3

where *q*_e_ is the adsorption amounts at equilibrium (mg/g), *C*_e_ is the equilibrium concentration (mg/L) in both models, *q*_m_ is the maximum adsorption capacity (mg/g), *K*_L_ (L/mg) is the Langmuir isotherm constant, *K*_F_ (L/mg) is the Freundlich parameter, and *n* is the constant describing the adsorption intensity. Generally, *n* > 1 means the adsorbent is favorable for adsorption, *n* = 1 predicts a linear adsorption, and *n* < 1 predicts an unfavorable adsorption.

**The related equations of adsorption** **kinetics**

The data are fitted to pseudo-first-order (Eq. S4) model, pseudo-second-order (Eq. S5) model, and intra-particle diffusion (Eq. S6) model:

$ln(\text{q}\text{e} -q\text{t} ) = ln\text{q}\text{e} - \text{k}\text{1} t$ Eq. S4

$t/q\text{t}= 1/(\text{k}\text{2} q_{e}^{2}) + t/\text{q}\text{e}$ Eq. S5

$q\text{t}=k\text{p}t\text{1/2}+C$ Eq. S6

where *q*_e_ (mg/g) represents the amount of adsorption at equilibrium, and *q*_t_ represents the amount of adsorption at time *t* (min). *k*_1_ (min ^−1^) is the rate constant of the pseudo-first-order model, *t* is the adsorption time (min). Here, the values of *k*_1_ and *q*_e_ were determined by linear fitting of ln(*q*_e_ − *q*_t_) to *t*. *k*_2_ [g/(mg min)] is the rate constant of the pseudo-second-order model. The values of *k*_2_ and *q*_e_ are determined by linear fitting of *t*/*q*_t_ to *t*. The values of *k*_p_ (the intra-particle diffusion constant (mg/g min^1/2^)) and *C* (thickness of the boundary layer (mg/g)) are determined by linear fitting of *q*_t_ to *t*^1/2^.

Fig. S1 (a) The adsorption kinetics of UIO-66-NH_2_ sample towards pentafluorophenol, (b) Data of adsorption kinetics fitted to the pseudo-first-order kinetic model, and (c) intra-particle diffusion model.

Fig. S2. The adsorption capacity of UIO-66-NH_2_-3 sample towards pentafluorophenol with the initial concentration of 2 mg/L, 4 mg/L, 6 mg/L, 8 mg/L, and 10 mg/L.

Fig. S3. the removal efficiency of pentafluorophenol by UIO-66-NH_2_-3 under various pH value.

Fig. S4. the Zr leaching of pentafluorophenol by UIO-66-NH_2_-3 under various pH value

Fig. S5. The adsorption capacity of UIO-66-NH_2_-3 towards pentafluorophenol in the presence of coexisting anions and organic molecules with two concentrations (50 mg/L and 100 mg/L).

Fig. S6. The (a) adsorption capacity and (b) Zr leaching of UIO-66-NH_2_-3 over five cycles.

Fig. S7. The (a) SEM, (b) FT-IR and (c) XRD spectra of fresh UIO-66-NH_2_-3 and used UIO-66-NH_2_-3

Fig. S8. The adsorption capacity of UIO-66-NH_2_-3 over five cycles in real water

**Supporting Tables**

Table S1. Porous characteristics of the UIO-66-NH_2_, UIO-66-OH, and UIO-66-COOH

| Samples | S_BET_ (m^2^/g) | Total pore Volume (cm^3^/g) | Average pore diameter (nm) |
| --- | --- | --- | --- |
| UIO-66-NH_2_ | 1200 | 0.675 | 8.54 |
| UIO-66-OH | 100 | 0.647 | 7.21 |
| UIO-66-COOH | 642 | 0.576 | 6.87 |

Table S2. Fitting parameters of the adsorption isotherms of UIO-66 with different groups samples towards to DMSO Langmuir and Freundlich models.

|  |  | Langmuir | | | | Freundlich | | | | |
| --- | --- | --- | --- | --- | --- | --- | --- | --- | --- | --- |
|  |  | *q*_m_ (mg/g) | | *K*_L_ (L/mg) | *R*^2^ | n | *K*_F_ (L/g) | | | *R*^2^ |
| DMSO | UIO-66-NH_2_ | 164.48 | 0.3504 | | 0.9761 | 6.032 | | 19.162 | 0.9074 | |
|  | UIO-66-OH | 105.8 | 0.1765 | | 0.9738 | 4.994 | | 24.276 | 0.8793 | |
|  | UIO-66-COOH | 39.5 | 0.1045 | | 0.9838 | 5.601 | | 38.302 | 0.9333 | |

Table S3. Fitting parameters of the adsorption kinetics of UIO-66-NH_2_ towards DMSO to pseudo-first-order model, pseudo-second-order model and intra-particle diffusion model.

|  | *C*_0_ (mg/L) | pseudo-first-order model | | | pseudo-second-order model | | | Intra-particle diffusion model | | |
| --- | --- | --- | --- | --- | --- | --- | --- | --- | --- | --- |
|  |  | *k*_1_ (min^−1^ ) | *q*_e_,_cal_ (mg/g) | *R*^2^ | *k*_2_×10^3^  [g/(mg min)] | *q*_e_,_cal_ (mg/g) | *R*^2^ | *C* | *k*_p_  [mg/(g min^1/2^)] | *R*^2^ |
| DMSO | 50 | 0.102 | 130.57 | 0.8364 | 1.01 | 165.4 | 0.9932 | 4.67 | 6.24 | 0.9748 |

Table S4. Fitting parameters of the adsorption isotherms of UIO-66-NH_2_ with different content samples towards to DMSO Langmuir and Freundlich models.

|  |  | Langmuir | | | | Freundlich | | | | |
| --- | --- | --- | --- | --- | --- | --- | --- | --- | --- | --- |
|  |  | *q*_m_ (mg/g) | | *K*_L_ (L/mg) | *R*^2^ | n | *K*_F_ (L/g) | | | *R*^2^ |
| DMSO | UIO-66-NH_2_-1 | 119.5 | 0.1004 | | 0.9761 | 7.258 | | 18.245 | 0.9112 | |
|  | UIO-66-NH_2_-2 | 165.8, | 0.1865 | | 0.9738 | 5.214 | | 25.674 | 0.8674 | |
|  | UIO-66-NH_2_-3 | 210.9 | 0.2375 | | 0.9838 | 6.321 | | 40.674 | 0.9247 | |

Table S5. Fitting parameters of the adsorption kinetics of UIO-66-NH_2_-3 towards DMSO to pseudo-second-order model.

|  | *C*_0_ (mg/L) | pseudo-second-order model | | |
| --- | --- | --- | --- | --- |
|  |  | *k*_2_×10^3^  [g/(mg min)] | *q*_e_,_cal_ (mg/g) | *R*^2^ |
| DMSO | 2.0 | 0.21 | 18.62 | 0.9954 |
|  | 4.0 | 0.57 | 36.54 | 0.9866 |
|  | 6.0 | 1.11 | 53.97 | 0.9957 |
|  | 8.0 | 1.37 | 71.35 | 0.9958 |
|  | 10.0 | 1.84 | 90.24 | 0.9932 |
